# Incorporation of causative quantitative trait nucleotides in single-step GBLUP

**DOI:** 10.1186/s12711-017-0335-0

**Published:** 2017-07-26

**Authors:** Breno O. Fragomeni, Daniela A. L. Lourenco, Yukata Masuda, Andres Legarra, Ignacy Misztal

**Affiliations:** 1 Edgar L. Rhodes Center for Animal and Dairy Science, University of Georgia, Athens, GA USA; 2 GenPhySE, INRA, INPT, INP-ENVT, Université de Toulouse, 31326 Castanet-Tolosan, France

## Abstract

**Background:**

Much effort is put into identifying causative quantitative trait nucleotides (QTN) in animal breeding, empowered by the availability of dense single nucleotide polymorphism (SNP) information. Genomic selection using traditional SNP information is easily implemented for any number of genotyped individuals using single-step genomic best linear unbiased predictor (ssGBLUP) with the algorithm for proven and young (APY). Our aim was to investigate whether ssGBLUP is useful for genomic prediction when some or all QTN are known.

**Methods:**

Simulations included 180,000 animals across 11 generations. Phenotypes were available for all animals in generations 6 to 10. Genotypes for 60,000 SNPs across 10 chromosomes were available for 29,000 individuals. The genetic variance was fully accounted for by 100 or 1000 biallelic QTN. Raw genomic relationship matrices (GRM) were computed from (a) unweighted SNPs, (b) unweighted SNPs and causative QTN, (c) SNPs and causative QTN weighted with results obtained with genome-wide association studies, (d) unweighted SNPs and causative QTN with simulated weights, (e) only unweighted causative QTN, (f–h) as in (b–d) but using only the top 10% causative QTN, and (i) using only causative QTN with simulated weight. Predictions were computed by pedigree-based BLUP (PBLUP) and ssGBLUP. Raw GRM were blended with 1 or 5% of the numerator relationship matrix, or 1% of the identity matrix. Inverses of GRM were obtained directly or with APY.

**Results:**

Accuracy of breeding values for 5000 genotyped animals in the last generation with PBLUP was 0.32, and for ssGBLUP it increased to 0.49 with an unweighted GRM, 0.53 after adding unweighted QTN, 0.63 when QTN weights were estimated, and 0.89 when QTN weights were based on true effects known from the simulation. When the GRM was constructed from causative QTN only, accuracy was 0.95 and 0.99 with blending at 5 and 1%, respectively. Accuracies simulating 1000 QTN were generally lower, with a similar trend. Accuracies using the APY inverse were equal or higher than those with a regular inverse.

**Conclusions:**

Single-step GBLUP can account for causative QTN via a weighted GRM. Accuracy gains are maximum when variances of causative QTN are known and blending is at 1%.

## Background

Initially, genomic selection used a large set of single nucleotide polymorphisms (SNPs) for genetic evaluation without the explicit identification of quantitative trait loci (QTL) [1]. SNP estimation coupled with variable selection or weighting is a way to improve accuracy by emphasizing regions with major genes, which is generally called Bayesian regression and we will use this term throughout the paper.

Those Bayesian methods could not be implemented directly for commercial populations, for which only a fraction of animals are genotyped. The methods were incorporated indirectly by using pseudo-observations and combining results with pedigree structure [2, 3]. Such a methodology called multistep is close to optimal only when pseudo-observations are very accurate (e.g., sires in dairy cattle or crop trials). When the structure of the genotyped dataset is more complex, problems such as double counting of contributions from pedigree and phenotypes, and preselection bias [4] reduce accuracy. SNP best linear unbiased predictor (SNP BLUP) is equivalent to genomic BLUP (GBLUP) or BLUP with a genomic relationship matrix (GRM) [2]. Single-step GBLUP (ssGBLUP), which is an extension of GBLUP, can incorporate pedigree, genomic, and phenotypic information jointly by using a relationship matrix that combines pedigree and genomic relationships [5]; an equivalent ssGBLUP based on SNP effects only has also been implemented [6]. Due to its simplicity and accuracy, ssGBLUP is now a method of choice for genomic evaluation in many livestock species.

When the number of genotyped animals is small, the use of Bayesian regression was found to increase accuracy of genomic prediction for many traits [7, 8]. However, as the number of genotyped animals increases, the improvement in accuracy becomes smaller or is zero. For example, VanRaden [2] reported that the improvement from non-linear predictions for milk yield in US dairy cattle was 4% in 2008 but dropped to 1% in 2011 [9]. In other words, the influence of the prior vanishes with larger amounts of data, a well-known property of Bayesian inference. A small improvement could be an artifact due to the use of non-coding SNPs. If all causative SNPs are identified, only those markers need to be fit in the model and the accuracy could approach 100%.

When the number of genotyped animals is very large, the computing costs of ssGBLUP, especially for inverting the GRM, could be prohibitive. Such costs could be reduced if the dimensionality of the genomic information is limited and exploited to reduce computations. VanRaden [2] found that the GRM has limited dimensionality and that blending of GRM with pedigree relationships (numerator relationship matrix, NRM) was required for numerical stability of GBLUP. Dimensionality of the GRM can be understood as the number of linearly independent genotypes that are present in the GRM. This dimensionality of the genomic information can be equally assessed by the eigenvalues of the GRM, the eigenvalues of the design matrix of SNP-BLUP, and the squares of singular values from singular value decomposition of the matrix of SNP content (matrix containing genotyped animals in the rows and each SNP genotype in the columns), which are all identical. Indirectly assuming limited dimensionality, Misztal et al. [10] proposed a method for the inversion of GRM called algorithm for proven and young (APY) based on the inversion of a small matrix of “core” animals, followed by a sparse expression for the other individuals. APY has a cubic computational cost for the size of the core subset but cost is only linear for the remaining animals. If the size of the core subset is not too large, APY can successfully invert GRM for millions of animals at a small cost. When tested in Holsteins, APY based on any core subset of more than 15,000 animals maximized the accuracy of genomic prediction [11]. APY was successfully used with several datasets that included up to 500,000 genotyped animals [12–14], which indicates that the dimensionality of the genomic information is indeed limited. Misztal [15] suggested that the dimensionality of the genomic information is proportional to effective population size (Ne). In simulations that involved populations with different Ne, accuracy was maximized when the number of animals in the core subset was equal to 4NeL, where L is genome length in Morgan [16]. However, accuracies decreased by less than 5% when the core subset size was equal to NeL. The number 4NeL (or NeL) is associated with the effective number of genomic segments, and was approximately 14,000 (3500) for Holsteins, 12,000 (3000) for Jerseys, 11,000 (2750) for Angus, and 4000 (1000) for pigs and broilers [17].

The concept of dimensionality of the genomic information, as described above, applies to generic GRM; however, it can also be applied to trait-specific or weighted GRM. If SNP selection for a specific trait results in only *n* SNPs being retained, the dimensionality cannot be greater than *n*. Subsequently, a trait-specific GRM that is created via SNP selection or GWAS is likely to have lower dimensionality than a generic GRM. Subsequently, the ratio of trait-specific to generic dimensionality could be an indicator of complexity of the trait. In particular, a low value of this ratio for a trait-specific GRM that results in the highest accuracy of GEBV would indicate that relatively few genes control this trait.

Recent advances in sequencing methodologies have renewed the interest in finding genes or QTN. If a trait is influenced by *n* QTN, the rank of the trait-specific genomic information (including GRM) is *n*, since only the QTN need to be used for the evaluation, and the accuracy of the genomic prediction reaches 100% if the dataset is large enough to estimate all QTN effects accurately. More realistically, if only a fraction of the causative QTN is identified, then both causative and non-causative SNPs must be used in the analyses. Some studies showed no improvement in accuracy of genetic evaluations when sequence data was included [18, 19], whereas other studies reported a small improvement [20–25]. Brøndum et al. [26] reported an important insight about the use of causative SNPs in genetic prediction i.e. they observed that including QTN with non-coding SNPs and using GBLUP or Bayesian regressions for the analyses did not result in any substantial increase in accuracy. However, accuracy increased when QTN were assigned more weight, in other words, higher a priori variance of their effects, to avoid these being heavily regressed towards zero like in SNP-BLUP. Thus, specific knowledge of those a priori variances is needed to correctly weight QTN.

If some causative QTN are identified, it would be useful to incorporate them in a simple analysis with increased gains in accuracy. The first goal of our study was to determine the properties of ssGBLUP when all or some QTN are identified and the second goal was to determine the dimensionality of genomic information when QTN are known and whether APY is applicable.

## Methods

### Heterogeneous SNP variances and weighted genomic relationship matrix

SNP-BLUP and GBLUP are equivalent models [2]. In particular, the breeding value is a linear function of SNP effects:

$${\mathbf{a}} = {\mathbf{Zs}},$$

where ${\mathbf{s}}$ is a vector of SNP effects, ${\mathbf{a}}$ is a vector of breeding values, and ${\mathbf{Z}}$ is a matrix of gene content, centered on the allele frequencies that are obtained from the entire genotyped population being evaluated. Assuming an equal distribution of SNP effects:

$$var\left( {\mathbf{s}} \right) = {\mathbf{I}}\sigma_{s}^{2} , var\left( {\mathbf{a}} \right) = {\mathbf{G}}\sigma_{a}^{2} = {\mathbf{ZZ}}^{\prime } \sigma_{s}^{2} ,$$

where $\sigma_{s}^{2}$ is the SNP variance, ${\mathbf{G}}$ is a genomic relationship matrix (GRM), and $\sigma_{a}^{2}$ is the additive variance. GRM can be derived directly from the a priori SNP variance as:

$${\mathbf{G}} = {\mathbf{ZZ}}^{\prime } \frac{{\sigma_{s}^{2} }}{{\sigma_{a}^{2} }}.$$

Assuming that the additive variance and gene frequencies are known, and under certain assumptions including Hardy–Weinberg and linkage equilibrium, the SNP variance is estimated as follows:

$$\sigma_{s}^{2} = \frac{{\sigma_{a}^{2} }}{{\mathop \sum \nolimits_{i}^{m} 2p_{i} q_{i} }},$$

so that based on [2]:

$${\mathbf{G}} = {\mathbf{ZZ}}^{\prime } \frac{{\sigma_{s}^{2} }}{{\sigma_{a}^{2} }} = \frac{{{\mathbf{ZZ}}^{\prime } }}{{\mathop \sum \nolimits_{i}^{m} 2p_{i} q_{i} }},$$

where $p_{i}$ is the allele frequency of the $i$-th SNP and $q_{i} = \left( {1 - p_{i} } \right)$. Allele frequencies were calculated using all genotypes in ${\mathbf{G}}$.

Assume a priori unequal SNP variances:

$$var\left( {\mathbf{s}} \right) = \left( {\begin{array}{*{20}l} {\sigma_{s,1}^{2} } \hfill & 0 \hfill & \ldots \hfill & 0 \hfill \\ 0 \hfill & {\sigma_{s,2}^{2} } \hfill & \ldots \hfill & 0 \hfill \\ \ldots \hfill & \ldots \hfill & \ldots \hfill & \ldots \hfill \\ 0 \hfill & 0 \hfill & \ldots \hfill & {\sigma_{s,n}^{2} } \hfill \\ \end{array} } \right),$$

where $\sigma_{s,i}^{2}$ is the variance of the $i$-th SNP effect and $n$ is the number of SNPs. Then, it is possible to use a SNP-BLUP with these variances [27] or, alternatively, GBLUP with a “weighted” genomic covariance matrix $Var\left( {\mathbf{a}} \right) = {\mathbf{Z}}var\left( {\mathbf{s}} \right){\mathbf{Z}}^{\prime }$. Specifically, GRM can include a diagonal matrix ${\mathbf{D}}$ of “weights”, such that:

$$Var\left( {\mathbf{a}} \right) = {\mathbf{Z}}var\left( {\mathbf{s}} \right){\mathbf{Z}}^{\prime } = \frac{{{\mathbf{ZDZ}}^{\prime } }}{{\mathop \sum \nolimits_{i = 1}^{m} 2p_{i} q_{i} }}\sigma_{a}^{2} = {\mathbf{G}}\sigma_{a}^{2} ,$$

where the factor $\sum\nolimits_{i = 1}^{m} {2p_{i} q_{i} }$ is introduced for compatibility with the current software so that for the unweighted GRM ${\mathbf{D}} = {\mathbf{I}}$ and $m$ is the number of SNPs. The contribution of locus $i$ to the covariance matrix ${\mathbf{G}}$ must be equal to its contribution in ${\mathbf{Z}}var\left( {\mathbf{s}} \right){\mathbf{Z}}^{\prime }$:

$$\varvec{z}_{i} \varvec{z}_{i}^{'} d_{i} \frac{1}{{\mathop \sum \nolimits_{i = 1}^{m} 2p_{i} q_{i} }}\sigma_{a}^{2} = \varvec{z}_{i} \varvec{z}_{i}^{'} \sigma_{s,i }^{2} .$$

Thus,

$$\sigma_{s,i}^{2} = d_{i} \frac{1}{{\mathop \sum \nolimits_{j = 1}^{m} 2p_{j} q_{j} }}\sigma_{a}^{2}, \quad {\text{and}} \quad d_{i} = \sigma_{s,i }^{2} \frac{{\mathop \sum \nolimits_{j = 1}^{m} 2p_{j} q_{j} }}{{\sigma_{a}^{2} }}.$$

In other words, $d_{i}$ is proportional to $\sigma_{s,i }^{2}$. The genetic variance in the population is $\sigma_{a}^{2} = \sum 2p_{i} q_{i} \sigma_{s,i }^{2}$, which means that all weights must average to 1. In practice, $\sigma_{s,i }^{2}$ are not available (or even estimated) and are often substituted by the squared effect of the SNP ($d_{i} \approx \hat{s}_{i}^{2} \frac{{\mathop \sum \nolimits_{j = 1}^{m} 2p_{j} q_{j} }}{{\sigma_{a}^{2} }}$). Because $\sum 2p_{i} q_{i} \hat{s}_{i}^{2}$ does not add up to the genetic variance of the population, $\sigma_{a}^{2}$, weights $d_{j}$ are, after estimation, standardized to sum to 1. Thus, in practice $d_{i}$ can be computed as equal to $\hat{s}_{i}^{2}$ and then scaled. Another approximation involves the squared effect of the SNP, weighted by the population heterozygosity ($d_{i} \approx 2p_{i} q_{i} \hat{s}_{i}^{2} \frac{{\mathop \sum \nolimits_{j = 1}^{m} 2p_{j} q_{j} }}{{\sigma_{a}^{2} }}$) [28], but this has no theoretical justification and gave poorer results in our study (not shown). Thus, here, the form $d_{i} \approx \hat{s}_{i}^{2} \frac{{\mathop \sum \nolimits_{j = 1}^{m} 2p_{j} q_{j} }}{{\sigma_{a}^{2} }}$ was used, by including either the estimated effect (for SNPs or QTN) or the true effect (of the QTN, in which case $\hat{s}_{i}^{2} = s_{i}^{2}$).

### Simulation

Using the software QMSim [29], we simulated a livestock population under selection for a single quantitative trait that has a heritability of 0.3. A historical population was generated by mutation and drift over 1000 generations, expanding from 1000 to 10,000 individuals, in order to create initial linkage disequilibrium (LD). For each replicate, 180,000 animals were simulated across 11 overlapping generations. Phenotypes were available for all animals in generations 6 to 10. For the first generation, 15,000 males and 15,000 females were simulated. A litter size of one individual was set resulting in 15,000 progeny in each generation, with a male to female ratio of 1:1. Sire and dam replacement rates of 20% were applied, animals were selected based on the highest estimated breeding values (EBV) estimated by BLUP at the end of each generation, and mating of selected animals was at random.

Genomic information was available only for animals in the last five generations. All animals with progenies were genotyped, i.e. 24,000 sires and dams. In addition, 5000 animals were randomly selected from the last generation to be genotyped. We simulated 10 chromosomes each 150 cM long and with evenly spaced 6000 SNPs, i.e. 60,000 SNPs in total. Each chromosome contained either 10 or 100 biallelic randomly located QTN (casual variants), i.e. 100 or 1000 QTN in total that are not included on the 60,000-SNP array. QTN effects were sampled from a gamma distribution with a shape parameter of 0.4 and scaled internally for a genetic variance of 0.3, and explained 100% of the genetic variance of the trait.

### Analysis

We used two methods for genetic evaluation: PBLUP and ssGBLUP. Both included 75,000 phenotypes in generations 6 to 10 and all pedigree information. The linear model was the same for all analyses and scenarios:

$${\mathbf{y}} = \mathbf{1}\upmu + {\mathbf{Wa}} + {\mathbf{e}},$$

where ${\mathbf{y}}$ is the observation vector, $\upmu$ is the mean, ${\mathbf{a}}$ is the vector of the animals’ additive effects, ${\mathbf{e}}$ is the vector of residuals, and ${\mathbf{W}}$ is the incidence matrix. Assumptions for residual effects were the same in all methods:

$${\mathbf{e}} \sim {\text{N}}\left( {\mathbf{0},{\mathbf{I}}\upsigma_{\text{e}}^{2} } \right),$$

where $\upsigma_{\text{e}}^{2}$ is the simulated residual variance, and ${\mathbf{I}}$ is an identity matrix with dimension equal to the number of animals.

The first method was PBLUP with ${\mathbf{a}} \sim {\text{N}}\left( {\mathbf{0},{\mathbf{A}}\upsigma_{\text{a}}^{2} } \right)$, where $\upsigma_{\text{a}}^{2}$ is the genetic additive variance and ${\mathbf{A}}$ is the numerator relationship matrix. The second method was ssGBLUP with ${\mathbf{a}} \sim {\text{N}}\left( {\mathbf{0},{\mathbf{H}}\upsigma_{\text{a}}^{2} } \right)$, where ${\mathbf{H}}$ is defined as in Legarra et al. [30] and its inverse is the same as in BLUP is [4]:

$${\mathbf{H}}^{ - 1} = {\mathbf{A}}^{ - 1} + \left( {\begin{array}{*{20}l} \mathbf{0} \hfill & \mathbf{0} \hfill \\ \mathbf{0} \hfill & {{\mathbf{G}}_{\text{b}}^{ - 1} - {\mathbf{A}}_{22}^{ - 1} } \hfill \\ \end{array} } \right),$$

where ${\mathbf{A}}_{22}^{ - 1} \varvec{ }$ is the inverse of the numerator relationship matrix for genotyped animals, and ${\mathbf{G}}_{\text{b}}$ is a “blended” GRM as described next.

Matrix ${\mathbf{G}}$ was constructed using different combinations of SNPs and weights: (a) unweighted with 60,000 non-coding SNPs; (b) unweighted with non-coding SNPs and the 100 or 1000 causative QTN; (c) as in (b) but with weights in ${\mathbf{D}}$ calculated based on genome-wide association studies (GWAS) using iterative ssGBLUP as in Wang et al. [31]; (d) as in (b) but unweighted for non-coding SNP ($d_{i} = c$, where $c$ was a constant equal to the smaller simulated QTN variance) and with weights based on true QTN effects as $d_{i} = s_{i}^{2} \frac{{\mathop \sum \nolimits_{j = 1}^{m} 2p_{i} q_{i} }}{{\sigma_{a}^{2} }}$; (e) unweighted using only 100 or 1000 causative QTN; (f–h) as (b–d) but using only 10% of the largest QTN; and (i) weighted by the true simulated variance using only 100 or 1000 causative QTN. Thus, QTN weights were proportional to $s_{i}^{2}$. Table 1 summarizes information about these scenarios. In an additional scenario, SNPs that are adjacent to causative variants received a weight equal to 0, while all other SNPs received the same constant for the polygenic effect, and causative SNPs received the simulated true effect as weight. The number of adjacent SNPs with weight equal to 0 started from 1 and increased until all non-coding SNPs had their weight set to 0.

**Table 1:** Parameters for the analysis of scenarios

Scenario	60 k SNPs	Causative QTN	Weights GWAS	Causative variances
(a)	Yes			
(b)	Yes	Yes		
(c)	Yes	Yes	Yes	
(d)	Yes	Yes		Yes
(e)		Yes		
(f)	Yes	Top 10%		
(g)	Yes	Top 10%	Yes	
(h)	Yes	Top 10%		Yes
(i)		Yes		Yes

‘60 k SNPs’ defines scenarios that included the simulated SNPs

‘Causative QTN’ defines scenarios that included all or the top 10% simulated causative variants

‘Weight GWAS’ defines scenarios that used weights from the iterative GWAS approach

‘Causative variance’ defines scenarios that used true simulated variance for QTL

Then, a scaled ${\mathbf{G}}_{0}$ was constructed as follows:

$${\mathbf{G}}_{0} = {\text{a}}{\mathbf{I}} + {\text{b}}{\mathbf{G}},$$

where constants ${\text{a}}$ and ${\text{b}}$ ensure equivalence of genomic and pedigree-based average relatedness and inbreeding [32], and ${\mathbf{I}}$ is an identity matrix with the same dimensions as ${\mathbf{G}}$. Because this ${\mathbf{G}}_{0}$ is not guaranteed to be positive definite [2], three alternative blended genomic matrices (${\mathbf{G}}_{\text{b}}$) were constructed from ${\mathbf{G}}_{0}$ as ${\mathbf{G}}_{\text{b}} = \left( {1 -\upalpha} \right){\mathbf{G}}_{0} +\upalpha{\mathbf{K}}$, where $\alpha$ is a blending factor and ${\mathbf{K}}$ is a positive definite matrix. We considered three cases: blending with either $\alpha = 0.05$ or $0.01$ of ${\text{A}}_{ 2 2}$, or with $\alpha = 0.01$ of the identity matrix. The inverse of ${\mathbf{G}}_{\text{b}}$ was obtained either by direct inversion or by APY [15]. In the latter case, the number of core animals was either (a) the number of the largest eigenvalues explaining 98% of the variance of ${\mathbf{G}}_{\text{b}}$, or (b) twice the number of simulated QTN.

The quality of predictions was assessed for the 5000 genotyped animals in the last generation. The accuracy was measured as the Pearson correlation between the genomic EBV (GEBV) and the simulated true breeding value (TBV). All calculations were done by using the BLUPF90 program suite [33], preGSf90 [34] to calculate the genomic matrices and postGSf90 for the GWAS [34]. All analyses were replicated 10 times.

## Results and discussion

**Fig. 1:**
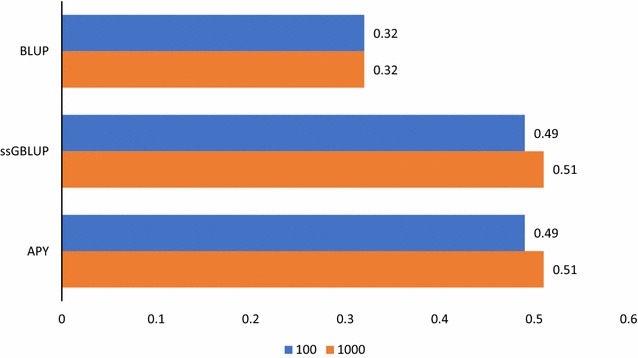
Accuracies of predictions with BLUP and ssGBLUP. Predictions with only pedigree information (BLUP) or genomic information using unweighted GRM derived from 60 k SNPs and a regular inverse (ssGBLUP), and as ssGBLUP but with the GRM inverse derived using APY. The number of causative QTN is 100 or 1000

**Fig. 2:**
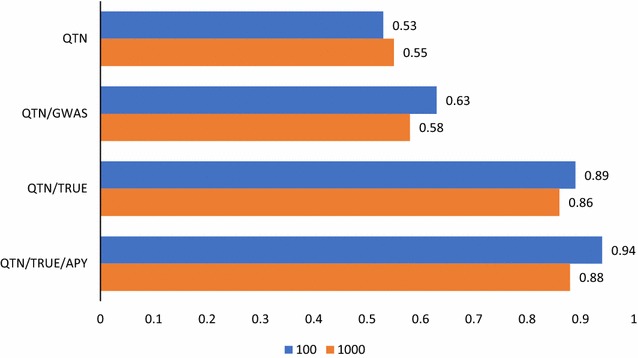
Accuracies of prediction with ssGBLUP including causative variants. Predictions with ssGBLUP with an unweighted GRM derived from 60 k SNPs and causative QTN and a regular inverse (QTN), as QTN but with a weighted GRM with weights derived from GWAS (QTN/GWAS), as QTN but with a GRM weighted by true QTN effects (QTN/TRUE), and as QTN/TRUE but with the APY inverse (QTN/TRUE/APY). The number of causative QTN is 100 or 1000

**Fig. 3:**
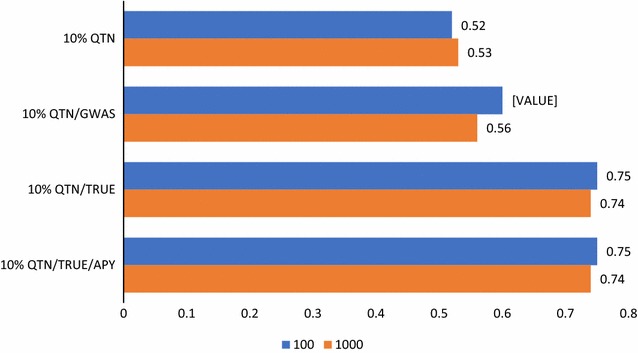
Accuracies of prediction with ssGBLUP including the top 10% causative variants. Predictions with ssGBLUP with an unweighted GRM derived from 60 k SNPs + the top 10% causative QTN and a regular inverse (10% QTN), as 10% QTN but with a weighted GRM with weights derived from GWAS (10% QTN/GWAS), as 10% QTN but with a GRM weighted by true QTN effects (10% QTN/TRUE), and as 10% QTN/TRUE but with the APY inverse (10% QTN/TRUE/APY). The number of causative QTN is 100 or 1000

**Fig. 4:**
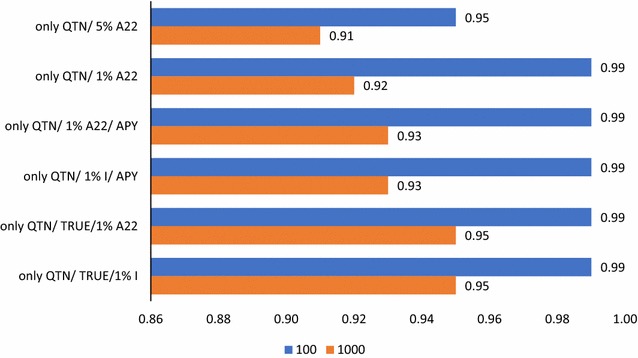
Accuracies of prediction with ssGBLUP including only causative variants. Predictions with ssGBLUP with an unweighted GRM with causative QTN only and a regular inverse with 5% blending by pedigree relationships (only QTN/5% ${\mathbf{A}}_{22}$), as only QTN/5% ${\mathbf{A}}_{22}$ but with 1% blending by pedigree relationships (only QTN/1% ${\mathbf{A}}_{22}$), as only QTN/1% ${\mathbf{A}}_{22}$ but with inversion by APY with the number of core animals equal to twice the number of QTN (only QTN/1% ${\mathbf{A}}_{22}$/APY), as only QTN/1% ${\mathbf{A}}_{22}$/APY but with blending of the identity matrix by 1% (only QTN/1% I/APY). Predictions with GRM weighted by true QTN effects were used with 1% pedigree relationship blending (only QTN/TRUE/1% ${\mathbf{A}}_{22}$) and 1% identity matrix blending (only QTN/TRUE/1% ${\mathbf{I}}$). The number of causative QTN is 100 or 1000

**Fig. 5:**
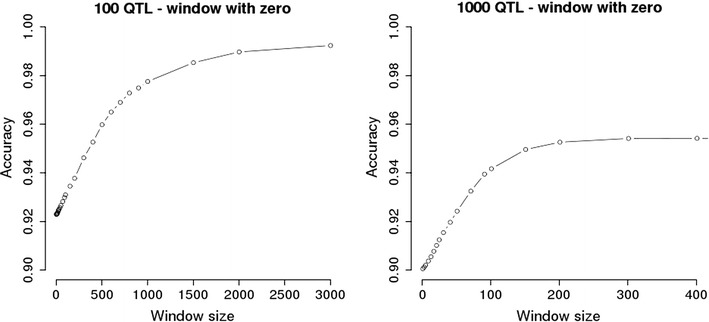
Accuracy of prediction with ssGBLUP without SNPs flanking QTN. Predictions with ssGBLUP with GRM derived from 60 k SNPs +causative QTN, weighted by the true simulated QTN effects and a constant for SNPs. SNPs flanking the causative variants had weights zeroed within the distance shown on the x axis

We observed very little difference between the realized accuracies across the replicates (≤0.01), and standard errors were <0.005, thus only the results of one replicate are shown. Accuracies obtained with different options are in Figs. 1, 2, 3, 4 and 5. LD was measured by r^2^ between adjacent SNPs with a mean (standard deviation) of 0.63 (0.06) across all chromosomes and generations.

### Including only non-coding SNPs

The accuracies obtained with PBLUP and ssGBLUP using only non-coding SNPs are in Fig. 1 and, as expected, were higher for ssGBLUP than for PBLUP. Accuracies were much lower than the value of 0.8 found for dairy cattle [35] because the number of phenotypes was much smaller but accuracies were close to those found for the broiler population for which a similar number of phenotypes was available [36]. Using the APY inverse with 16,000 randomly selected core animals resulted in the same accuracies as using the regular inverse. When an unweighted GRM was used to obtain the APY inverse, the optimum number of core animals was close to the number of the largest eigenvalues in the GRM that explained 98% of the variance [16], which in this case was close to 16,000.

### Including causative QTN

Figure 2 presents the accuracies obtained when using non-coding SNPs and causative QTN together. Including causative QTN in the unweighted GRM increased accuracies by 0.04, which is similar to the 2.5% increase in reliability reported by VanRaden et al. [25]. Karaman et al. [37] found that, as in Bayesian regressions, GBLUP partially accounts for QTL regions, in particular for very large datasets because the variances of the SNP effects constitute prior information that vanishes as the amount of data increases. Using weighted GRM with weights obtained by GWAS as described by Wang et al. [31], the accuracy increased further, by 0.10 for the data with 100 QTN and by 0.05 with 1000 QTN. This increase was higher with 100 QTN because these have larger effects, and because there are fewer effects to be estimated by the model. Using GWAS for weighting SNP effects seems to have a limited success due to the structure of LD [17, 38]. GWAS as used in this study is relatively simple; in BayesR or BayesRC, several sets of prior variances are available, with the largest set being potentially useful for identifying causative QTN [19, 22]. When creating the GRM by using true effects for causative QTN with small variances for the non-coding SNPs, accuracies increased substantially, i.e. by 0.36 with the 100 QTN data and 0.31 with the 1000 QTN data, as compared to the unweighted GRM including the causative variants. This confirms the assertion of Brøndum et al. [26] who reported that for accuracy to increase substantially with causative QTN, it is necessary to weight them differently. When the previous analysis was repeated with the APY inverse, accuracies increased even further, to 0.94 and 0.88, respectively. As accuracies approach 1 in the analyses that fully exploit all causative QTN, increases in accuracy with the APY inverse must be due to a decrease in noise from the non-coding SNPs. VanRaden et al. [25] obtained on average a 2.5% increase in reliability by incorporating potential causative SNPs while removing adjacent SNPs. Since one QTN generates a multi-SNP response [31, 39, 40], its incorporation in the analyses allows the removal of spurious effects of adjacent SNPs.

### Analyses with the top 10% causative QTN

Identifying all causative QTN and their weights is unrealistic, and Fig. 3 presents accuracies for scenarios similar to those above but including only the top 10% causative QTN. Compared to the scenario including all causative QTN, considering only the top 10% resulted in a decreased accuracy, as expected. The reduction was small with unweighted GRM, larger with weights via GWAS, and largest with the true weights of causative SNPs. Using the APY inverse does not improve the accuracy as in scenarios that include all QTN, because the non-coding SNPs are not redundant anymore since they are proxies for the 90% missing causative QTN.

### Analysis with causative QTN only

To investigate how blending of the GRM affects the accuracy with causative QTN, we conducted analyses using GRM calculated from QTN assuming equal weights and different blending factors (Fig. 4). While accuracies close to 1.00 were expected, the computed accuracies with blending factors of 5% and (1%) with the pedigree relationships (${\mathbf{A}}_{22}$) were equal to 0.95 and 0.91 and (0.99 and 0.92) with the 100 and 1000 QTN data, respectively. Using the APY inverse with the number of core animals equal to twice the number of QTN resulted in the same accuracy as with the 100 QTN data and increased by 0.01 with the 1000 QTN data. Accuracies obtained with a 1% blending factor with the identity matrix or ${\mathbf{A}}_{22}$ were identical.

When all causative QTN are known, blending with pedigree relationships only adds noise and is done for numerical stability. Blending at a 5% factor adds more noise than blending at 1%, and blending with the identity matrix may be slightly superior. The lower accuracy that is obtained with the 1000 QTN data can be explained by the use of an unweighted GRM. In SNP-BLUP, a large amount of data overwhelms the priors of variances when the number of SNPs is small (say 100) but less when it is larger (say 1000). Since SNP-BLUP and GBLUP are equivalent [2, 41], the same applies to GBLUP or ssGBLUP. When all causative SNPs are known, blending of GRM as used for the APY inverse is for numerical stability only. One way to eliminate blending is to estimate genomic breeding values by using a reduced model, which includes only the core animals in the equations and derives predictions for the remaining animals as linear functions of the core animals [42]. However, the optimal number of core animals is not an exact parameter, since varying the number of core animals by a factor of more than 2 (from 95 to 99% of the explained variance in GRM) changed the realized accuracy by 0.01 only [16].

### Removing SNPs around causative QTN

Assigning zero as a weight for SNPs around causative variants increased the accuracy, until the weight of all non-causative SNPs was set to 0, which caused accuracies to reach the maximum of 0.99 for the 100-QTL scenario and 0.95 for the 1000-QTL scenario (Fig. 5). The shapes of the two curves were very similar, but scales differed i.e. in the 1000-QTL scenario, accuracy increased by a factor 10. This increase was observed because there were 10 times more SNPs with a zero weight in the scenario with more QTL. The shape of the curves showed that the difference in accuracy is bigger when the genomic segments with weights set to 0 are shorter. This can occur for two reasons. First, most of the non-causative SNPs had a weight set to 0 when the number of SNPs set to 0 was equal to 600 in the 100-QTL scenario or 60 in the 1000-QTL scenario; thus, random spacing of QTL could still allow a few SNPs to have a weight different from 0. Second, removing the SNPs that are located near causative variants is actually equivalent to removing SNPs that are “hitchhiking” because of LD. This is especially true for the SNPs that are located near QTL with a larger effect. Similar results were reported by VanRaden et al. [25] who found that removing SNPs around Manhattan plots peaks improved the resolution for potential causative variants in dairy cattle data. In drosophila, Ober et al. [43] showed that accuracy of phenotype prediction of phenotypes increased when non-causative SNPs were excluded from the analysis, but the pattern of accuracy fluctuated considerably, probably because of the small sample size.

### Dimensionality of the genomic relationship matrix

Table 2 shows the number of eigenvalues required to explain a certain percentage of variance of GRM with various options. For unweighted and unblended GRM, the number of eigenvalues required to explain 90, 95 and 98% variance was about 8500, 12,000, and 17,000, respectively, with little difference between 100 and 1000 QTN datasets. According to Pocrnic et al. [16, 17], the optimal dimensionality of the genomic information—for prediction—corresponds to the number of eigenvalues associated with 98% of variance in GRM, and linked those values to the number of independent chromosome segment (ICS). While the GRM is not full rank, the NRM is full rank. In theory, the number of ICS depends on the effective population size and the length of genome but not on the number of QTN [44]. A blending factor of 5% with ${\mathbf{A}}_{22}$ increased the number of eigenvalues by 10 to 15%. Increasing the blending factor with ${\mathbf{A}}_{22}$ makes the blended ${\mathbf{G}}$ better conditioned numerically although the amount of information is not increased.

**Table 2:** Number of eigenvalues explaining 90, 95 or 98% of the variance for genomic relationship matrices

Option	Number of eigenvalues					
	100 QTN			1000 QTN		
	90% eigenvalue	95% eigenvalue	98% eigenvalue	90% eigenvalue	95% eigenvalue	98% eigenvalue
60 k	8496	12,185	16,978	8502	12,192	16,984
60 K-BL5	9553	13,787	19,111	9560	13,796	19,120
60 K-GWAS3	4571	7537	13,139	4757	7704	13,230
60 K-QTN-BL5	9553	13,788	19,112	9563	13,806	19,136
60 k-QTN-BL5-TRUE^d^	76	1803	5093	469	1942	5140
60 k-QTN10-BL5-TRUE^a,b,d^	4054	8972	15,886	7482	13,320	19,918
60 K-QTN-BL5-GWAS3	4082	7084	12,880	4627	7594	13,186
QTN	88	94	98	793	872	930
QTN-BL5^c^	94	122	7639	863	980	7925
QTN-BL1^c^	89	95	127	806	888	995

Options used to construct the genomic relation matrix: 60 k non-coding SNPs (60 k), all causative QTN (QTN), the top 10% causative SNPs (QTN10), blending at 5% (BL5) or 1% (BL1), weighted by the 3rd iteration of the single-step GWAS (GWAS3), and weighted by true QTN effects (TRUE) for datasets with 100 or 1000 causative QTN

^a^10 eigenvalues explained 76% of the variance of ${\mathbf{G}}$ for the 100-QTN scenario

^b^100 eigenvalues explained 71% of the variance of ${\mathbf{G}}$

^c^Eigenvalues after number of QTN (100 or 1000) had values approaching 0 (below 10E−4)

^d^Simulated true weights for QTN and a constant equal to the minimum QTN value for SNPs

With GRM weighted by GWAS, the dimensionality was reduced especially at the 90% level. The reduction was bigger with fewer QTN, which indicated lower complexity of the trait as expected, but this difference was small. This could be due to limited efficiency of the method used for GWAS in this study. This method [31] estimates variances of SNP effects jointly, as opposed to sequentially in Bayesian methods, as squares of the SNP effects. Subsequently, the method is inefficient for QTL with small effects. Possible solutions include limiting the changes of variances from round to round as in NonlinearA [2], or setting the lower bound on the variance as in FastBayesA [45].

When the GRM was constructed using the QTN information only, the number of eigenvalues required to explain 90, 95 and 98% variance was close to the number of simulated QTN, especially for the scenario with 100 QTN. QTN were distributed randomly, and likely, QTN in large LD to adjacent QTN contributed little information, with more such QTN for the 1000-QTN scenario.

In a population with a different structure, QTN may be in LD with each other, and thus this number is expected to be smaller. Blending increased the dimensionality, especially at the 98% level. While this increase was at most 30% with a 1% blending factor, the increase was up to 8 (1000 QTN) and 77 times (100 QTN) with the 5% blending factor. While the extra dimensionality added noise, it made the matrix more stable to explicit inversion.

The numbers of eigenvalues obtained with the 10% top QTN are in between those obtained with no causative SNPs and with only causative SNPs. In general, the dimensionality of unweighted GRM could be equal to the number of ICS or close to 4NeL and the dimensionality of GRM constructed with causative QTN only would be equal to the number of those QTN or smaller (if some causative QTN have very little effect or are in LD). With GRM uniformly weighted for SNPs (with SNP weights accounting for a small proportion of the total genetic variance) and with true variances for all or the top 10% causative QTN, intermediate numbers of eigenvalues will be obtained.

## Conclusions

Information on causative QTN can be included in single-step GBLUP via a weighted GRM. To obtain a high accuracy of prediction, the matrix has to be constructed using realistic weights for the causative QTN, by possibly eliminating non-coding SNPs that are located close to causative QTN, and with very little blending with pedigree information, i.e. the minimum required for stability. Use of the APY algorithm for inversion of GRM results in increased or similar accuracy as with the regular inverse but at much reduced cost, regardless of the inclusion of SNPs, QTN, or both. Finally, the dimensionality of the genomic information is roughly the number of independent chromosome segments for unweighted GRM, the number of causative QTN for GRM weighted with their exact weights, and in between with a fraction of causative QTN or with GRM using weights from GWAS.
