# Erratum to: Incorporation of causative quantitative trait nucleotides in single-step GBLUP

**DOI:** 10.1186/s12711-017-0341-2

**Published:** 2017-08-24

**Authors:** Breno O. Fragomeni, Daniela A. L. Lourenco, Yutaka Masuda, Andres Legarra, Ignacy Misztal

**Affiliations:** 1 Edgar L. Rhodes Center for Animal and Dairy Science, University of Georgia, Athens, GA USA; 2 GenPhySE, INRA, INPT, INP-ENVT, Université de Toulouse, 31326 Castanet-Tolosan, France

## Erratum to: Genet Sel Evol (2017) 49:59 DOI 10.1186/s12711-017-0335-0

After publication of this paper [1], it was noted that the author’s name was spelt incorrectly. The author’s name should be spelt as: Yutaka Masuda.

The original article was corrected.
